# Effects of diffuse light on the gross ecosystem primary productivity of a winter wheat (*Triticum aestivum* L.) cropland in northern China

**DOI:** 10.1038/s41598-024-65279-8

**Published:** 2024-06-22

**Authors:** Xueyan Bao, Xiaomin Sun

**Affiliations:** 1 Agricultural School, Inner Mongolia Minzu University, Tongliao, 028000 China; 2 Key Laboratory of Ecosystem Network Observation and Modeling, Institute of Geographic Sciences and Natural Resources Research, Chinese Academy of Sciences, Beijing, 100101 China

**Keywords:** Diffuse light, Ecosystem photosynthesis, Agroecosystem, Canopy light interception, Modeling, Biophysics, Plant sciences, Ecology, Environmental sciences

## Abstract

Diffuse light is produced by clouds and aerosols in the atmosphere. Exploring the effects of diffuse light on ecosystem productivity is important for understanding the terrestrial carbon (CO_2_) cycle. Here, 2 years of gross ecosystem primary productivity (GEP) from a (winter) wheat cropland in China was assessed using eddy covariance technology to explore the effects of diffuse photosynthetic active radiance (PAR) on wheat GEP. Wheat GEP increased significantly and positively along with diffuse PAR. In addition, wheat GEP was significantly affected by total PAR, air temperature, and vapor press deficit in different diffuse PAR fraction (fDIF) change stages. Because significant autocorrelations existed among the controlling factors, a path analysis was used to quantify the effects of diffuse light on GEP. Diffuse PAR was the primary and secondary importance factors affecting GEP with direct path coefficients of 0.54 and 0.48, respectively, in different fDIF change stages. A multilayer canopy model revealed that the middle and lower canopy levels intercepted more light when diffuse PAR increased. This resulted in the photosynthetic enhancement of middle and lower canopy levels, which contributed approximately 65% and 35%, respectively, to the increase in photosynthesis for the entire canopy (~ 30.5%). Overall, our study provided new evidence regarding the importance of diffuse light for CO_2_ uptake in agroecosystems, which is important for predicting the responses of ecosystem CO_2_ budgets to future climate-related light changes.

## Introduction

Solar radiation provides energy for plant photosynthesis and is an important factor influencing plant carbon (CO_2_) assimilation^1–4^. Terrestrial carbon assimilation rates for a leaf-level response to sunlight increases nonlinearly with solar radiation until leaves are light saturated^5,6^. However, at the vegetative canopy scale, the photosynthetic response to solar radiation becomes more complex than on a leaf scale because of leaf arrangement and distribution within a canopy^4,7,8^. Recent evidence in worldwide observational networks indicates that there are existing coherent periods and regions that show the prevailing “dimming” and “brightening” of solar radiance^9–13^. Thus, investigating the dependence of ecosystem-level CO_2_ production on sunlight is important for predicting terrestrial carbon cycles under the background of climate-related light changes.

When sunlight penetrates the Earth’s atmosphere, it interacts with clouds and aerosols, creating diffuse light^14^. The presence of clouds and aerosols, along with the increase in diffuse light intensity or fraction, enhance ecosystem carbon uptake^6,15–19^, forming the fertilization effect of diffuse light. Zhang et al.^20^, indicated that the canopy carbon uptake over a maize cropland increases during cloudy days compared with clear days. Cheng et al.^16^, found a significant linear positive relationship between canopy photosynthesis and diffuse light intensity. Based on global simulations, Mercado et al.^6^, demonstrated that increases in the diffuse light fraction reaching the canopy from 1960 to 1999 may have enhanced the global carbon sink by approximately 25%.

Several hypotheses have been proposed by the plant science community to explain the effects of diffuse light on ecosystem photosynthesis^16^. The most important may be that the more even and deep distribution of light in a canopy^21^. In detail, under direct-light conditions, plant leaves in the canopy receive light from a single direction. Thus, the leaves of the lower canopy are shaded and receive less light because of the light interception of upper leaves if the canopy is close. In comparison, the canopy is illuminated from multi-directions under diffuse-light conditions; consequently, light penetrates deeper and is distributed more evenly in the canopy. Consequently, leaves that were previously shaded now receive more light, and light interception by the canopy increases^4^. However, only a few studies have tested this hypothesis. Urban et al.^21^, indicated that the leaves in the middle and lower spruce canopy levels assimilate more CO_2_ on cloudy days compared with on sunny days because of the even vertical distribution of light throughout the canopy. Williams et al.^4^, found that the proportion of deep shade within the canopy is significantly greater under direct conditions compared with under diffuse conditions, resulting in greater photosynthesis under the latter. However, these studies explored the mechanism by measuring leaves at different canopy layers to represent parts, i.e. upper, middle, or lower canopy levels. Because one canopy level commonly includes several leaf layers, using the photosynthesis value of only one leaf layer to represent that of a particular canopy level may generate uncertainties.

In nature, diffuse-light intensity changes are not separate processes, and these changes are often inextricably linked with total light, air temperature (T_a_) and vapor depress deficit (VPD)^11^. The variations among meteorological factors may lead to different responses in ecosystem photosynthesis to a diffuse light increase. Some studies found nonobvious incremental alteration in ecosystems photosynthesis or even reduced ecosystems photosynthesis under diffuse-light conditions due to reduced total light on cloudy or aerosol days^22–25^. The different response patterns of ecosystem photosynthesis to diffuse-light changes may reflect the different sensitivities of different ecosystems to the variation in diffuse light. Kanniah et al.^3^, illustrated that the sensitivity of ecosystem photosynthesis to diffuse-light is correlated with canopy structural characteristics, such as the leaf area index (LAI), leaf angle orientation, leaf clumping factor and canopy height. Because terrestrial ecosystems are diverse in their canopy traits, more site-scale studies on the relationship between ecosystem photosynthesis and diffuse light should be carried out to collect valuable information.

There are also some issues regarding the effect of diffuse light that need further exploration, e.g. to what extent is diffuse light important for canopy photosynthesis? The answer is important because if diffuse light contributes very little to photosynthetic variations, then the effects of diffuse light on an ecosystem may not be significant, even though it can promote canopy photosynthesis. To date, few studies have quantified the effects of diffuse light and determined its relative importance for ecosystem carbon uptake. For example, one study on a forest ecosystem indicated that diffuse light is not as important as total light and vapor pressure deficit by using an artificial neural networks method^26^. Another study reported that the contribution of diffuse light to canopy photosynthesis over a soybean cropland is much smaller than other factors^16^. However, these studies did not consider the correlations among different drivers when quantifying the effects of diffuse light.

In this study, the eddy covariance (EC) technique combined with a modeling method were used to calculate the over-canopy variation in the gross ecosystem primary productivity (GEP) and to simulate the within-canopy microclimate conditions and photosynthetic rate in a (winter) wheat (*Triticum aestivum* L*.*) crop in northern China from 2010 to 2012. Our aim was to comprehensively explore the effects of diffuse light on wheat GEP, including the pattern of response and the magnitude and mechanism of the effects by (1) analyzing the response of GEP to a diffuse-light increase; (2) quantifying the effects of diffuse light on GEP and determining its relative importance; and (3) exploring the mechanisms underlying the effects of diffuse light. We hypothesized that increased diffuse light will enhance wheat GEP as a result of more light being intercepted by the canopy.

## Materials and methods

### Study area and experimental measurements

The field experiment was conducted over a (winter) wheat (*Triticum aestivum* L.) cropland at the Luancheng Agroecosystem Experimental Station (37° 50′ N, 114° 40′ E; elevation: 50.1 m above sea level) in Hebei Province, China, during 2010–2012. The cultivars were ‘Kenong 199’ and ‘Kenong 1066’, which were provided by Hebei Academy of Agricultural of Forest Sciences, in the two growing seasons. The climate of the region is semi-humid, with a long winter (November to the following February) and a short spring (March to April)^27^. The long-term (from 1990 to 2008) mean annual temperature and precipitation were 12.8 ℃ and 485 mm, respectively. Wheat was sown in October and harvested in June. The greatest canopy height was approximately 1.0 m. The maximal LAIs for wheat were approximately 4.1 m^2^ m^−2^ and 3.9 m^2^ m^−2^ at the heading growing stage in 2011 and 2012, respectively.

The ecosystem CO_2_ and heat fluxes between the biosphere and atmosphere were measured using EC technology. The EC monitoring system consisted of a three-dimensional sonic anemometer (Model CSAT 3, Campbell Scientific Inc., USA) to monitor fluctuations in vertical wind velocity (ω’) and an open-path and fast-response infrared gas analyzer (Model LI-7500, LI-COR Biosciences Inc., Lincoln, NE, USA) to monitor the fluctuations in the CO_2_ and water vapor concentrations (q’)^27^. The net ecosystem CO_2_ exchange (NEE; mg CO_2_ m^−2^ s^−1^), latent heat flux (LE; W m^−2^ s^−1^), and sensitivity heat flux (H; W m^−2^ s^−1^) were calculated on line using the covariance between ω’ and q’. Along with the flux measurements, variations in global radiation, net radiation, total photosynthetic active radiation (400–700 nm; hereafter denoted as PAR), air temperature (T_a_), surface soil temperature, relative humidity, soil water content (SWC), precipitation, and soil heat flux were also measured. A data logger collected the raw data at a rate of 10 Hz and stored them as 30 min averages. Details of other instruments used for taking measurements and data collection were described previously^28^.

The raw flux and meteorological data subjected to correction, screening, and rejection processes^5,29^. Detailed information regarding data processing were previously described^28^.

### GEP estimation and light-response curve

GEP is the difference between ecosystem respiration (ER) and NEE (GEP = ER − NEE). Nighttime ER (equal to nighttime NEE because there is no photosynthesis during the nighttime) gaps were interpolated using the Lloyd & Taylor model^30^. Daytime ER was estimated using the method proposed by Reichstein et al.^31^. Detailed information regarding ER interpolation was published in Bao et al.^28^.

To obtain the entire GEP time series for whole growing seasons, the gaps in daytime NEE was filled. The gaps having time intervals of less than 2 h were filled using the linear interpolation method. Gaps of longer than 2 h were filled using the marginal distribution sampling method, in which the “gaps” could be filled on the basis of relationships between flux and environmental factors^29^.

### Diffuse PAR estimation

At the Luancheng site, a direct measurement of the diffuse component of solar radiance was lacking; consequently, the strength of diffuse light was estimated on the basis of the clearness index (CI) and the diffuse component of global solar radiation (S_d_). Many studies have used this model-based method to estimate diffuse light (fraction) or the cloudiness index^20,32,33^. According to Gu et al.^34^, the CI is the ratio of the global solar radiation (S, W m^−2^) received by the earth’s surface to the extraterrestrial irradiance at a plane parallel to the Earth’s surface (S_e_, W m^−2^) as follows:

1 $$\text{CI = }\frac{\text{S}}{{\text{S}}_{\text{e}}},$$

2 $${\text{S}}_{\text{e}}\text{=}{\text{ S}}_{\text{sc}}\text{[1+0.003cos(360}{\text{t}}_{\text{d}}\text{/365)]sin}\beta \text{,}$$

3 $${\text{sin}}\beta \text{ = sin }\varphi \, \cdot \text{ sin }\delta \text{ + cos }\varphi \, \cdot \text{ cos }\omega \text{,}$$

where S_sc_ represents the solar constant (1370 W m^−2^), t_d_ represents the day of the year, $\beta$ represents the solar altitude angle, $\varphi$ represents the degree of latitude, $\delta$ represents the declination of the sun, and $\omega$ represents the time angle^34^.

In this study, diffuse light refers to the diffuse component of photosynthetic active radiance (PAR). The diffuse fraction of total PAR (fDIF) and diffuse PAR were calculated using the following relationships^35^:

4 $${\text{fD}}{\text{IF}}\text{=}\frac{\left[\text{1+0.3}\left(\text{1} - {\text{q}}^{2}\right)\right]{\text{q}}}{\text{1+(1-}{\text{q}}^{2}\text{)}{\text{cos}}^{2}\text{(90-}\beta \text{)}{\text{cos}}^{3}\beta },$$

5 $$\text{q} = \frac{{\text{S}}_{\text{d}}}{{\text{S}}_{\text{e}}\cdot {\text{CI}}},$$

Because CI is the ratio of S to ${\text{S}}_{\text{e}}$,

6 $$\text{q} = \frac{{\text{S}}_{\text{d}}}{\text{S}}.$$

When $$0\le \text{CI}\le 0.3,$$ restrain: $$\text{q}\le 1.0,$$

7 $$\text{q}=1.02-0.254\text{CI}+0.0123\text{sin}\left(\beta \right).$$

when $$0.3<\text{CI}<0.78,$$ restrain: $$0.1\le \text{q}\le 0.97,$$

8 $$\text{q}=1.400-1.749\text{CI}+0.177\text{sin}\left(\beta \right).$$

when $$\text{CI}\ge 0.78,$$ restrain: $$\text{q}\ge 0.1,$$

9 $$\text{q}=0.486\text{CI}-0.182\text{sin}\left(\beta \right).$$

$${\text{PAR}}_{{{\text{dif}}}} = {\text{ PAR}}_{{{\text{tot}}}} \times {\text{ fDIF}}.$$

where PAR_dif_ represents diffuse PAR, PAR_tot_ represents total PAR, and fDIF represents diffuse PAR fraction.

### Path analysis

To quantify the effects of diffuse PAR on GEP and determine its relative importance, a path analysis method was used in this study. The path analysis quantifies the effects of independent variables to dependent variables and considers the correlations among controlling (independent) factors^36,37^. First, Pearson’s correlation analysis was used to determine the factors significantly controlling GEP and the existence of significant relationships among them. Second, a path analysis was conducted to establish controlling paths and the corresponding coefficients according to the Pearson’s correlation coefficients. The effects of independent variables are indicated by the direct coefficients of the relationships between the independent variables and GEP. The relative importance of one factor can be determined by comparing the direct coefficients of different controlling factors. The software packages used to conduct Pearson’s correlations and the path analysis were SPSS (ver. 19.0 SPSS Inc., Chicago, IL, USA) and Amos (ver. 5.0; SPSS Inc.), respectively.

### Biophysical multilayer canopy model

The EC technology only measures the above-canopy CO_2_ flux. To obtain the CO_2_ exchange rate within the canopy, we used a biophysical multilayer canopy model documented by Baldocchi and Wilson^9^. This model computes the biosphere–atmosphere exchange of water vapor, CO_2_, and sensible heat flux, as well as the microclimate, within and above the canopy on an hourly timescale. The model consists of micrometeorological and physiological modules. The former computes the leaf and soil energy exchange and scalar concentration profiles throughout the canopy. Environmental factors that were computed using the micrometeorological module drive the physiological modules that compute leaf photosynthesis. The model was driven by external variables that were measured above the canopy. The environmental inputs included incident PAR, air temperature, wind speed, relative humidity, and CO_2_ concentration. Plant structural variables included the LAI, leaf angle orientation, leaf clumping factor, and canopy height. During the study period, the leaf area and plant height were relatively constant. The LAI almost reached its maximal value of approximately 3.9 m^2^ m^−2^ during the study period. The average plant height was 76 cm. Leaf transmittance in the bottom canopy was approximately 0.3^38^. The averaged leaf angle was approximately 59°. The clumping factor characterizes the spatial distribution of leaves or needles within a canopy. Data on the clumping factor for wheat were not recorded during the study period; therefore, the model used a clumping factor = 1, which indicates a homogeneous canopy with a random dispersion of leaf area, as suggested by Tang et al.^39^. The entire canopy was divided into 10 layers on average, from canopy top to ground surface, by the model, and the meteorological conditions (mainly referring to the incident PAR) and photosynthetic rate of each layer were simulated. Mean values of the photosynthetic rate and PAR levels in layers 1–3, 4–7, and 8–10 were used to represent the upper, middle, and lower levels of the canopy, respectively.

### Model validation

**Figure 1:**
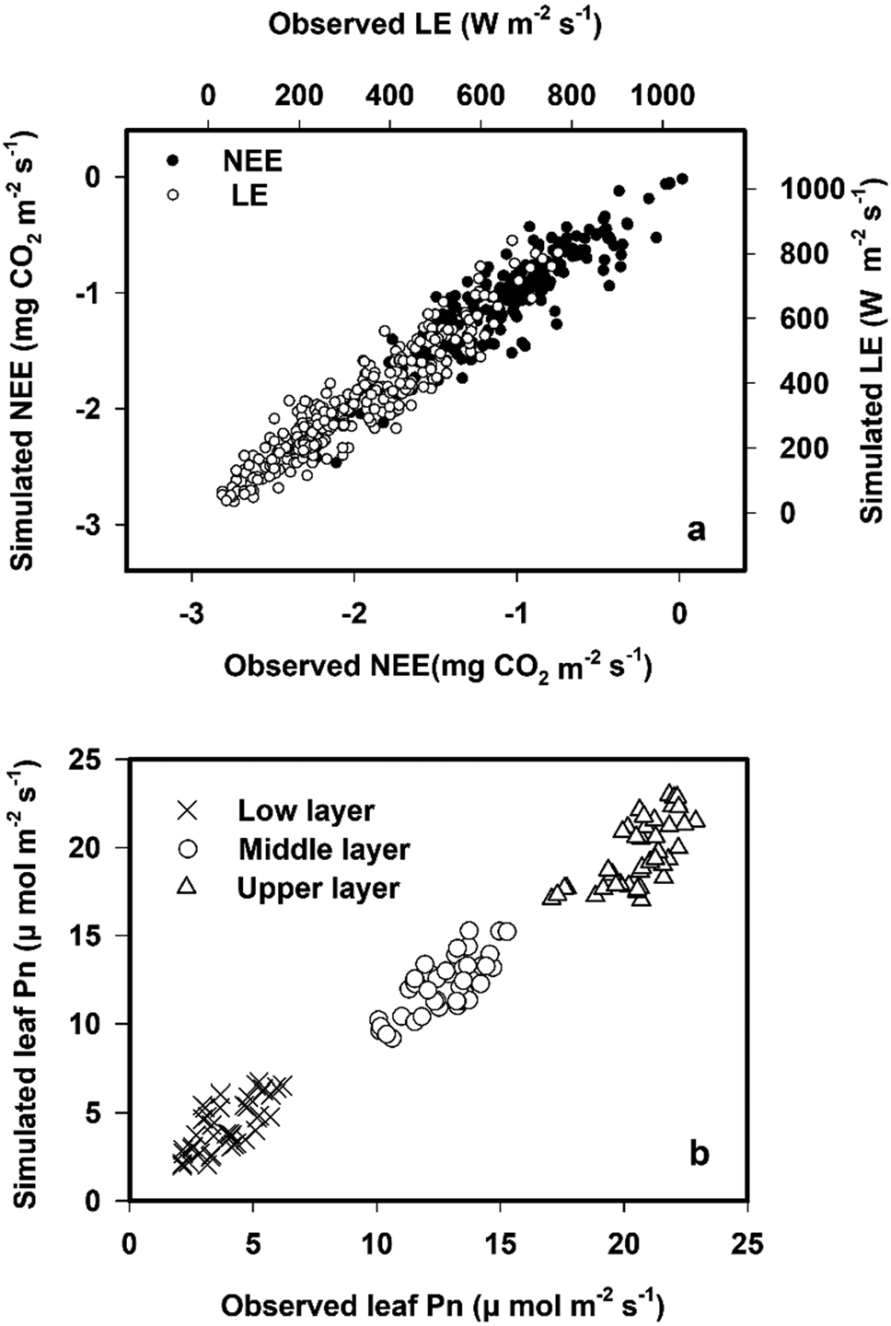
Validation for the multilayer canopy model through observed above-canopy fluxes (**a**) and leaf photosynthetic rates (Pns) in different canopy layers (**b**). All the correlations were significant at the level of *p* = 0.01.

The biophysical multilayer canopy model was validated by the observed above-canopy fluxes and the measured within-canopy photosynthesis rate during the target periods. The above-canopy fluxes (CO_2_ and water) were measured using EC technology (Section “Study area and experimental measurements”). The within-canopy photosynthesis rate was measured using Li-6400XT (LI-COR Biosciences Inc.). In the target crop field, five wheat plants that were not growing in the field boundary were randomly selected, and the instantaneous photosynthetic rates of the upper-, middle-, and lower-layer leaves of each plant were measured between 10:00 am and 11:00 am every 3 to 4 days under clear and cloudless sky conditions during the study period. Here, five plants were selected because this ensured enough samples for the photosynthetic measurements and allowed the plants to be measured at the same time, which minimized the difference in photosynthetic rates among plants resulting from the light change. When verifying the model, the simulated values of different canopy layers corresponding to the photosynthetic measured times were selected to be compared with the measured values. The slope of a linear relationship between simulated versus observed data (*k*) was used to describe the validation results. The multilayer canopy model predicted CO_2_ and LE flux above the canopy levels well, with *k* ≈ 1 (Fig. 1a). There were also significant linear relationships between simulated and observed leaf photosynthesis values in different canopy layers, with *k*s of 0.87, 0.91, and 0.89 for the lower, middle, and upper layers, respectively (Fig. 1b). These relationships demonstrated that the prediction results of the model were reliable.

### Analyzed periods

During growing seasons, the leaf areas of crops typically change markedly along with plant growth. To minimize the impact of leaf area changes on CO_2_-exchange processes, data from April to middle May for wheat were selected. This period covered mainly the stages from flowering to seed-filling for wheat, during which the plants grew relatively steadily and the changes in the LAI were not dramatic. In addition, due to different solar zenith angles having varied effects on the responses of ecosystem CO_2_ exchanges to light or the role of diffuse PAR^16^, daytime data were collected from 10: 00 am to 14:00 pm during the selected growing period. The GEP values estimated based on observed and unfilled NEE data were used.

### Statement

All the plant and field experiments were conducted in accordance with relevant institutional, national, and international guidelines and legislation.

## Results

### Weather conditions and GEP variations

**Figure 2:**
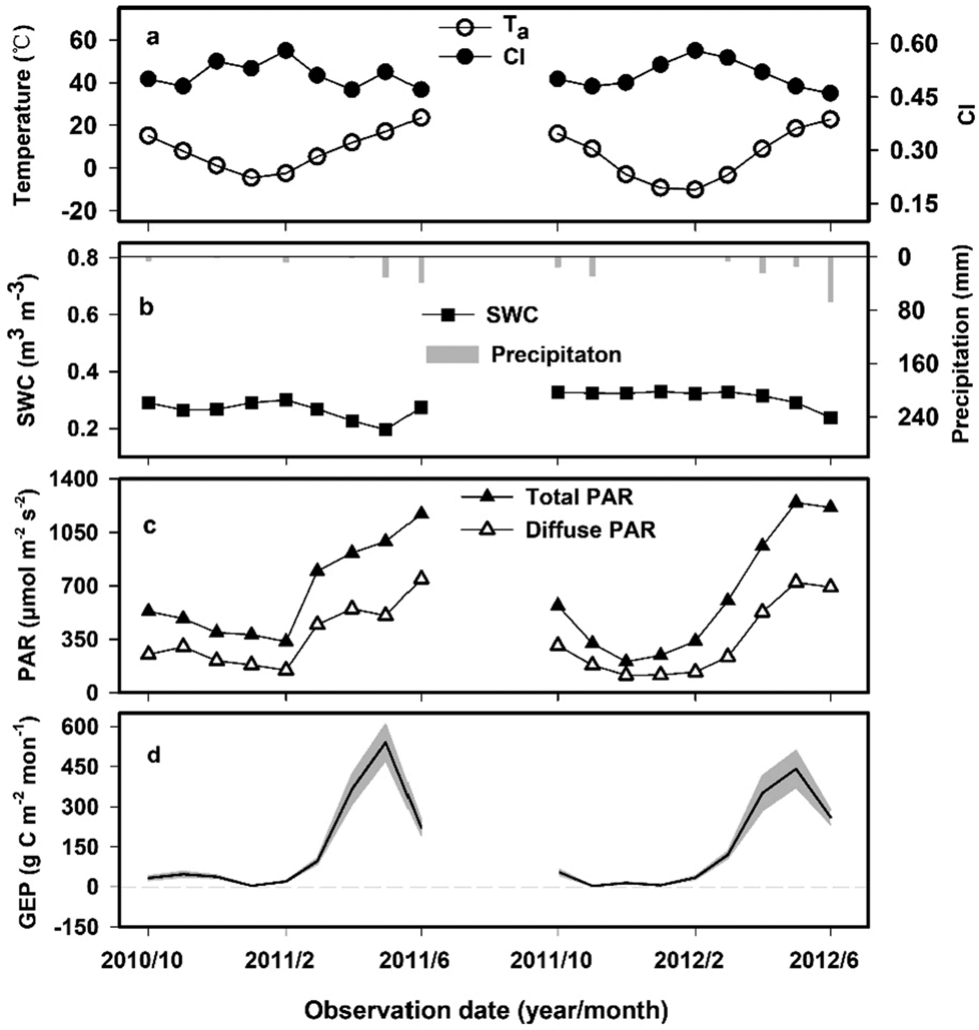
The seasonal variations in (**a**) monthly mean air temperature (T_a_, ℃), and clearness index (CI); (**b**) soil water content (SWC, m^3^ m^−3^) and monthly summed precipitation (mm); (**c**) monthly mean total PAR (μmol m^−2^ s^−1^) and diffuse PAR; and (**d**) monthly summed GEP (g C m^−1^ mon^−1^) for wheat during 2010–2012. The gray area in (**d**) represents the uncertainty of the monthly values calculated in accordance with the method described by Bao et al.^22^.

The seasonal variations in meteorological factors and GEP are shown in Fig. 2. For each growing season, the minimal monthly mean T_a_ value occurred in the following January, and then, the values increased rapidly until harvest time (Fig. 2a). Monthly mean VPDs exhibited a variation trend similar to that of T_a_ (data not shown). The rainfall during the wheat growing season was less than during the rest of the year and it was mainly concentrated in May and July. The surface soil moisture conditions showed a gentle change (Fig. 2b). CIs varied obviously among the months. They tended to reach maximum values in February, indicating that the sky during this period was clearest (Fig. 2a). Solar radiation and its diffuse portion showed similar change trends (Fig. 2c). The total PAR and diffuse PAR values were low during winter and then began to increase gradually after February.

The daily GEP of wheat was close to zero from the sowing date in October to the reviving stage in the following February (Fig. 2d). In spring, the daily GEP began to increase rapidly and reached its maximum value in May, after which it decreased because of plant senescence.

### Effects of diffuse PAR and its effect magnitudes

**Figure 3:**
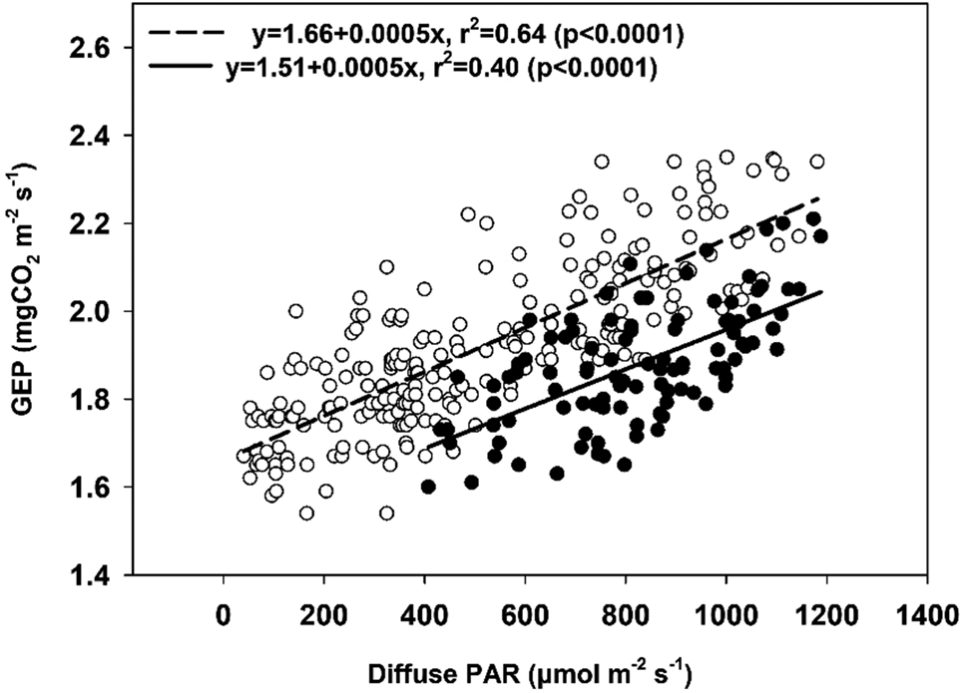
Response of GEP to diffuse PAR in a wheat cropland. The solid and open circles indicate the data pairs corresponding to fDIF change stage from a minimal to moderate values when diffuse PAR reaches it maxima (Stage 1) and fDIF change stage from moderate to maximal values (Stage 2), respectively. The values presented in the panel are the mean values of 2 years.

**Figure 4:**
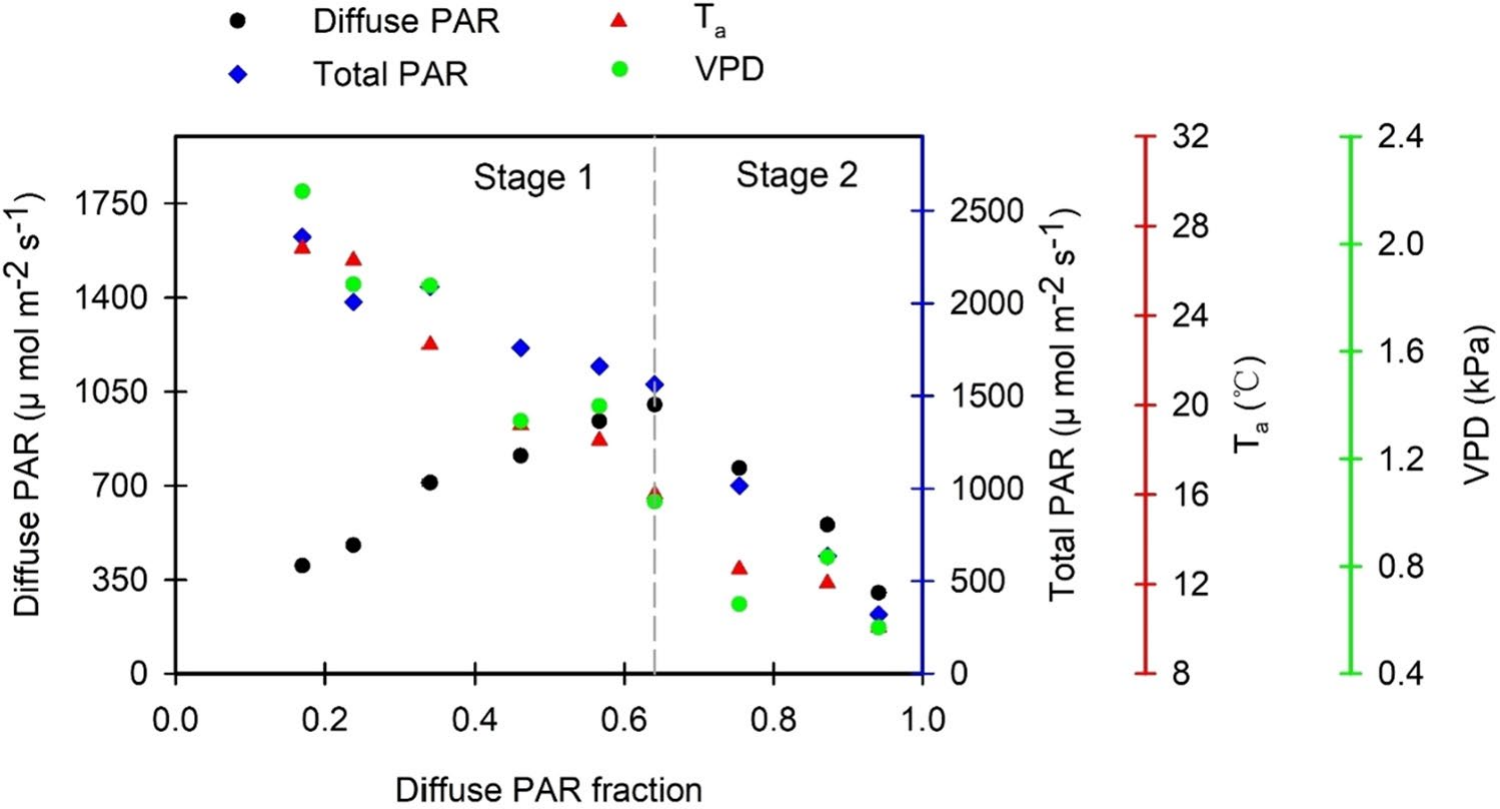
Relationships between diffuse PAR fraction (fDIF) and diffuse PAR, total PAR, T_a_, and VPD. Stage 1 refers to fDIF change stage from minimal to moderate values when diffuse PAR reaches it maxima, and Stage 2 refers to fDIF change stage from moderate to maximal values. The values presented in the panel are the mean values of 2 years.

The relationship between diffuse PAR and wheat GEP is shown in Fig. 3. Wheat GEP increased significantly along with diffuse PAR linearly during the study period. In detail, GEP increased with diffuse light during the stage in which the diffuse PAR fraction (fDIF) increased from minimal to moderate values. This was when diffuse PAR reached its maxima (denoted as fDIF change Stage 1) and increased from moderate to maximal values (denoted as fDIF change Stage 2). The different fDIF change stages were separated based on the relationship between diffuse PAR and fDIF (Fig. 4).

**Figure 5:**
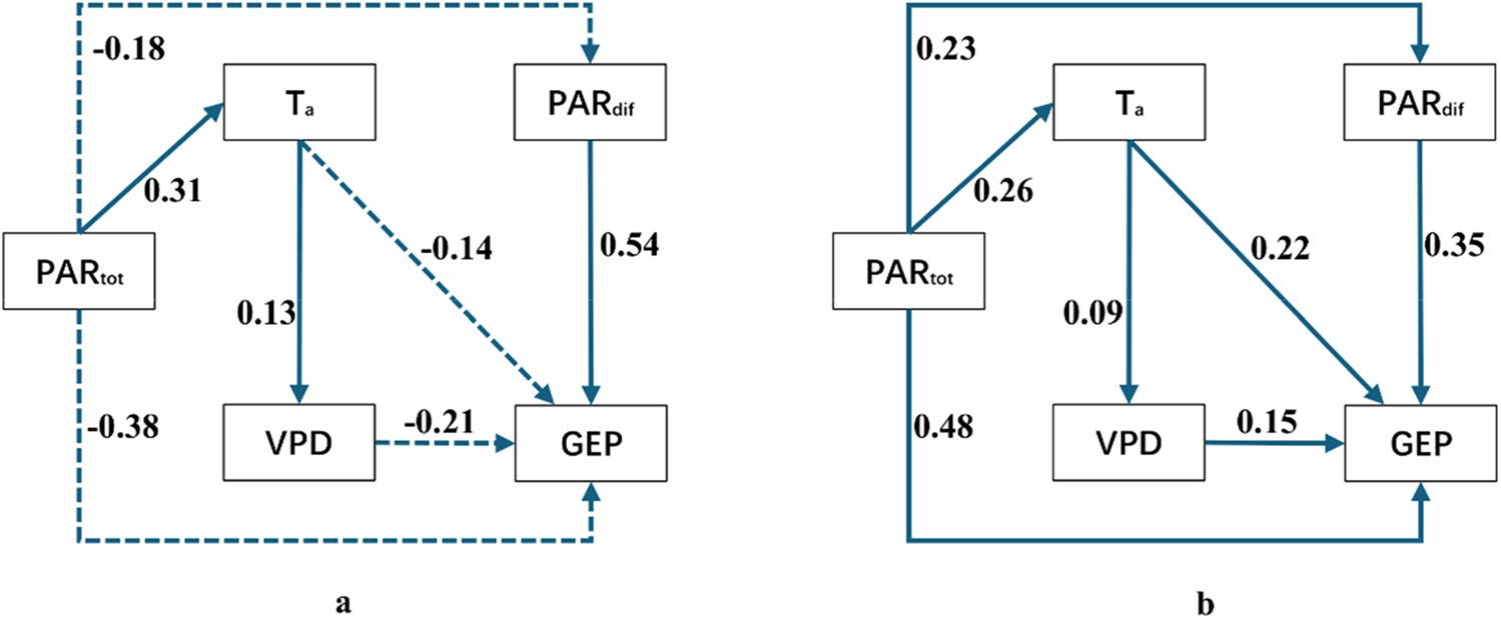
Path analysis of the effects of different controlling variables on variations in GEP in fDIF change Stage 1 (**a**) and Stage 2 (**b**) of the wheat cropland in this study. The arrows represent controlling paths from one variable to another variable. Numbers near arrows represent the path coefficients. The solid and dashed lines represent positive and negative correlation between different variables, respectively.

When diffuse PAR changed, other environmental factors also varied. Pearson’s correlation analysis indicated that total PAR, T_a_, and VPD each had negative effects on GEP (Table 1). This suggested that variation in wheat GEP in Stage 1 was jointly affected by diffuse PAR, total PAR, T_a_, and VPD. The path analysis (Fig. 5) showed that diffuse PAR was the most important factor affecting GEP in Stage 1 because the direct path coefficient of diffuse PAR was 0.54, which was the largest among the affecting factors. Total PAR was the second important factor, followed by T_a_ and VPD. In Stage 2, GEP was positively and significantly correlated with diffuse PAR, total PAR, VPD, and T_a_ (Table 1). The path analysis for this stage indicated that total PAR was the most important factor affecting GEP, with a direct path coefficient of 0.48, whereas diffuse PAR was the secondary important factor, with a direct path coefficient of 0.35, followed by T_a_ and VPD.

**Table 1:** Pearson’s determination coefficients (*r*^2^) of the relationships between half-hourly wheat GEP and the controlling factors in different fDIF change stages.

	Variables	T_a_	VPD	PAR_tot_	PAR_dif_	GEP
Stage 1	T_a_	1				
	VPD	0.692**	1			
	PAR_tot_	0.552*	0.369*	1		
	PAR_dif_	−0.424*	−0.375*	−0.431*	1	
	GEP	−0.465*	−0.417**	−0.404*	0.405*	1
Stage 2	T_a_	1				
	VPD	0.681**	1			
	PAR_tot_	0.508*	0.521*	1		
	PAR_dif_	0.492**	0.463**	0.380*	1	
	GEP	0.505*	0.426*	0.464*	0.643**	1

The analysis was conducted using the unfilled half-hour data of the study period for wheat.

* and ** denote the correlations at significant levels of 0.05 and 0.01, respectively. Stage 1 and Stage 2 refer to the fDIF change stage from minimal to moderate values when diffuse PAR reached it maxima and the stage from moderate to maximal values, respectively.

### Canopy light interception

**Figure 6:**
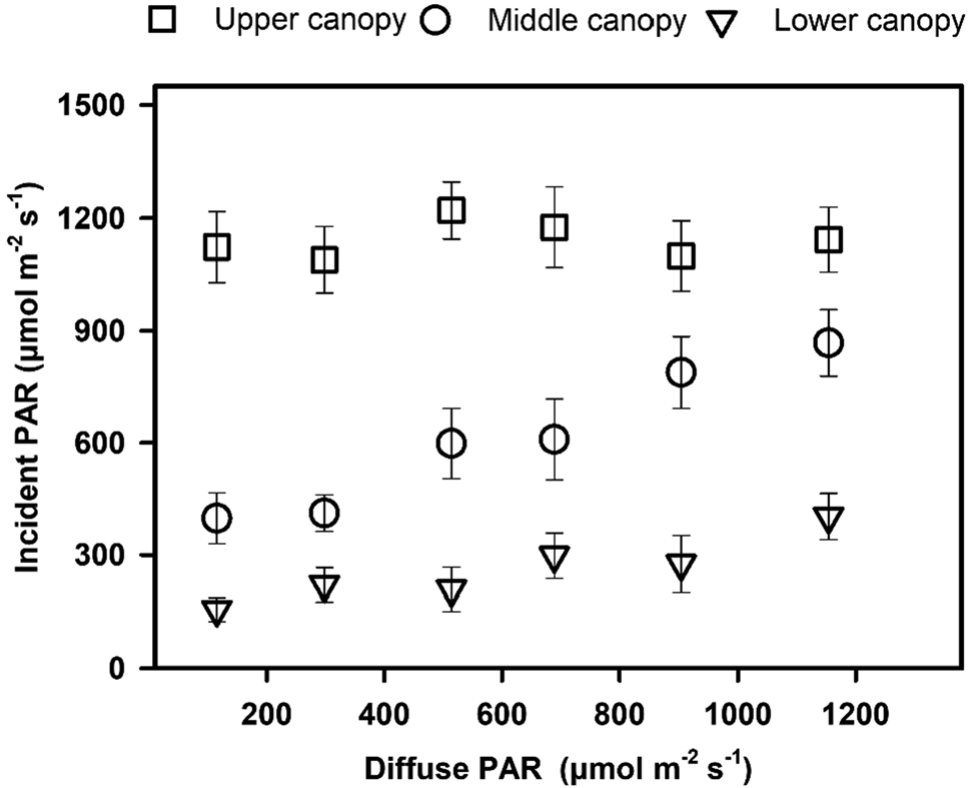
Response of simulated incident PAR in the upper, middle, and lower canopy levels to diffuse PAR divided into bins of 200 μmol m^−2^ s^−1^. The error bars indicate the standard deviations of incident PAR for each diffuse PAR bin. The incident PAR in the middle and lower canopy levels increased linearly with diffuse PAR, with determination coefficients of 0.96 (P < 0.01) and 0.86 (P < 0.05), respectively.

**Figure 7:**
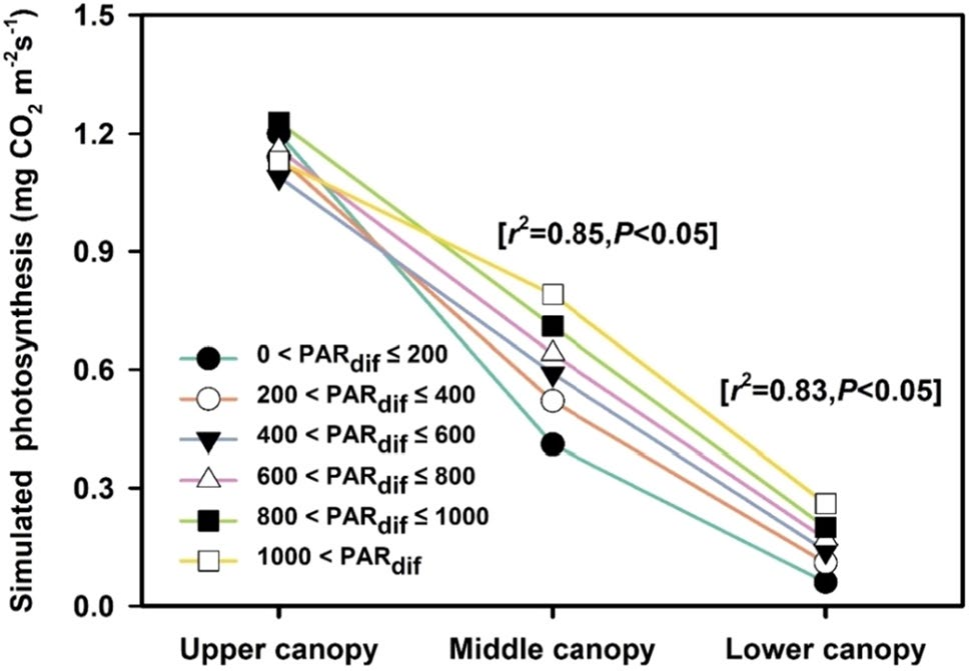
Gross photosynthetic rate for upper, middle, and lower wheat canopy levels simulated using a multiple canopy model. The averaged values over diffuse PAR bins of 200 μmol m^−2^ s^−1^ are presented. *r*^2^ represents the determine coefficient of the linear regression equation between the simulated photosynthetic rate and diffuse PAR for different canopy levels; *P* represents the significance of the correlations. The percentage in the parentheses is the ratio of the change in the photosynthesis of the corresponding canopy level to the total change in the photosynthesis of the entire canopy when diffuse PAR increased from the minimum to maximum value. PAR_dif_, diffuse PAR.

This study indicated that diffuse PAR produced positive effects on GEP during two fDIF change stages. To illustrate the mechanisms for the GEP enhancement due to the diffuse PAR increase, the incident light and photosynthetic rate within the canopy were simulated by the canopy model at varied diffuse PAR levels. The simulated incident PAR in the upper canopy was almost constant as diffuse PAR increased, whereas the simulated incident PAR in the middle and lower canopy levels increased significantly along with diffuse PAR (Fig. 6). This indicates that the incident light within a canopy is distributed more deeply and that the within canopy intercept increases along with diffuse PAR. The light distribution within the canopy caused vertical variations in the photosynthetic rate within the canopy. As shown in Fig. 7, the simulated photosynthetic rate of the upper canopy did not differ significantly under different diffuse PAR levels, whereas the photosynthesis in the middle and lower canopy levels was enhanced significantly as diffuse PAR increased, with determination coefficients of 0.85 and 0.83 for the middle and lower canopy levels, respectively. When diffuse PAR increased from its minimal to maximal level, the entire canopy photosynthesis (represented as the sum of photosynthetic rate for three canopy levels) increased by approximately 30.5%. The middle and lower canopy levels contributed approximately 65% and 35% to this increase, respectively, indicating that as diffuse PAR increased, the within canopy, especially the middle and lower levels, intercepted more light, leading to photosynthetic increases in these canopy levels and, consequently, the photosynthesis of the entire canopy.

## Discussion

### Environmental factors

Wheat GEP increased significantly along with diffuse PAR throughout the whole diffuse PAR change process, thereby confirming our initial hypothesis regarding the response pattern. This response of GEP to diffuse PAR indicated that GEP reached its maxima under moderate fDIF conditions and decreased when fDIF increased to the maxima or decreased to the minima because of the relationship between fDIF and diffuse PAR (Fig. 4). Our results were consistent with previous studies. For three different types of forest sites in China, Zhang et al.^40,41^, reported that the maximal CO_2_ uptake rates occurs at an intermediate fDIF level. Yang et al.^32^, also indicated a similar trend in GEP with increasing fDIF in two typical croplands in China. However, using observational data and modeling approaches, Alton et al.^24^, found a lower canopy photosynthesis under moderate fDIF conditions compared with under lower fDIF conditions, for 38 Flux sites covering several plant functional types. This difference between our study and previous studies indicates that some ecosystems are not sensitive to diffuse light increases but may be more sensitive to total light, and this insensitivity to diffuse light may relate to canopy properties such as the LAI, leaf optical parameters, and leaf angle^11,18^. For example, terrestrial vegetation with high LAIs tend to be more sensitive to increases in diffuse PAR because as the plant canopy becomes denser with leaves, which means that the canopy has greater LAI, canopy transmission to lower canopy level decreases, leading to limited radiation in the middle and lower canopy levels. Thus, the canopy photosynthesis would be more sensitive to diffuse PAR compared with a canopy having a low LAI^11^. Wolfahrt et al.^1^ also showed that grass systems with LAIs of more than 4 m^2^ m^−2^ show significant increases in net CO_2_ uptake under cloudy conditions compared with clear sky condition, whereas the net carbon uptakes of grasses with intermediate (2–4) to low (< 2) LAIs do not increase significantly.

By quantifying the effects of different factors on GEP variation through a path analysis, diffuse PAR was determined to be the predominant driver in fDIF change Stage 1, whereas total PAR was the most important factor affecting GEP in fDIF change Stage 2. These results indicate that light availability played predominant roles in affecting the ecosystem canopy photosynthesis in this wheat cropland. This supports the statements of Mercado et al.^6^ and Kanniah et al.^3^, who indicated that solar radiance is the primary driver of plant photosynthesis. However, our conclusion was not consistent with that of Cheng et al.^16^, who also used above–canopy measurements and reported that diffuse PAR explains only approximately 5% of ecosystem photosynthesis variations, thus having a very small effect on ecosystems in a rainfed soybean cropland. These inconsistent results may be due to the different analysis methodologies between the two studies. The study of Cheng et al.^16^, used multiple linear equations to quantify the effects of diffuse PAR, which did not consider the effects of total PAR and did not consider the correlations among controlling factors.

T_a_ and VPD typically impact photosynthesis by influencing photosynthetic enzyme activity levels^1^ and leaf stomatal behavior^40–42^, respectively. An increase in T_a_ below or above a threshold may promote or inhibit plant photosynthesis. The latter occurs because of stomatal closure caused by a high level of VPD that typically co-varies with temperature. The simple correlation analysis in our study indicated that GEP increased along with T_a_ and VPD in fDIF Stage 2, indicating that the weather conditions favored crop growth, with little water stress occurring. However, T_a_ and VPD appear to have small effects on GEP variations because their direct path coefficients were lower than those of total PAR and diffuse PAR. This result is consistent with those of previous studies in which temperature and VPD were found to play nonprimary roles in affecting plant productivity^15,23,26,43^.

### Mechanism of diffuse PAR effect

Using the combination of over-canopy flux measurements and the modeling method, our study indicated that as diffuse PAR increased, the middle and lower canopy levels intercepted more incident light, leading to increases in their photosynthesis, thereby confirming our hypothesis regarding the increase in light interception within the canopy due to deeper and more uniform light distribution when diffuse light increases. However, we did not find a significant increase in incident light in the upper canopy (Fig. 6), mainly because the incident PAR values at low diffuse PAR levels were the averages of higher incident light produced under low-fDIF conditions and lower incident light produced under high-fDIF conditions. The averaged incident values under the two extreme fDIF conditions may be close to the values under intermediate-fDIF conditions.

Although this study has revealed that the enhancement of canopy photosynthesis along with diffuse PAR is related to more light being intercepted by the canopy, the canopy photosynthetic response to diffuse light might be caused by other reasons. Previous studies proposed that diffuse light tends to weaken photoinhibition in sunlit leaves at the top of the canopy^44^, and thus, it increases entire-plant photosynthesis. In the current study, it was unclear whether strong light (under low diffuse PAR conditions) depressed GEP in the upper canopy because the GEP values corresponding to low diffuse PAR caused by clear sky and by heavy cloud conditions were averaged. Even when photosynthetic depression under strong light is found, the chemical reactions and enzymatic activities correlated with photosynthesis at the leaf scale should be analyzed without destroying the natural state of the vegetation to determine whether the photosynthetic decline is related to photoinhibition. Thus, to fully understand the affecting mechanism, research on correlations between photosynthetic physiological and ecological processes and light climate changes at cellular, leaf, and canopy scales are needed in the future.

### Further implications

Our study showed that when fDIF increased from minimum to intermediate levels, with diffuse PAR increasing but total PAR decreasing, and wheat GEP increased, indicating that the wheat planted in this site was more sensitive to diffuse PAR change than to total light. However, some ecosystems were less sensitive to diffuse PAR changes because the GEP of these ecosystems were not enhanced noticeably or were even depressed during the diffuse PAR increase process^22,23^. Owing to the spatial variability and biodiversity levels of agroecosystems, future studies should be conducted on a wide range of cropland types at site scale to determine which croplands are sensitive to diffuse light. Based on this information, it is valuable to analyze what canopy structure features they have in common, and whether there are spatial distribution patterns in the sensitivity across continents or even the globe. The answers to these questions are of great significance for accurately predicting the CO_2_ budget dynamics in farmland ecosystems under the background of climate-related light changes.

## Conclusions

In this study, we found that wheat GEP increased significantly along with diffuse PAR when the effects of crop growth and solar elevation angle were excluded. We quantified the effects of diffuse PAR and determined its relative importance because diffuse PAR was not the only factor affecting wheat GEP. A path analysis indicated that diffuse PAR was the primary factor and the secondary importance factor affecting GEP during different fDIF change stages. The mechanisms of diffuse light effects on canopy photosynthesis were tested using a modeling method. As diffuse PAR increased, the canopy, especially at the middle and lower canopy levels, intercepted more light, leading to photosynthetic increases in middle and lower canopy levels, and, consequently, the photosynthesis of the entire canopy was enhanced. Future studies should analyze the sensitivities of varied ecosystems in different regions to diffuse light and the multiple response mechanisms of ecosystem CO_2_ exchange to diffuse light change.

## Data Availability

The datasets generated during and/or analysed during the current study are available from the corresponding author upon reasonable request.
